# A calendar-aging-resistant aqueous battery

**DOI:** 10.1093/nsr/nwag304

**Published:** 2026-05-25

**Authors:** Zhuo Yang, Dongliang Chao, Dongyuan Zhao, Wanhai Zhou

**Affiliations:** Laboratory of Advanced Materials, Aqueous Battery Center, Shanghai Key Laboratory of Molecular Catalysis and Innovative Materials, Collaborative Innovation Center of Chemistry for Energy Materials, Shanghai Wusong Laboratory of Materials Science, State Key Laboratory of Porous Materials for Separation and Conversion, College of Smart Materials and Future Energy, Fudan University, China; Laboratory of Advanced Materials, Aqueous Battery Center, Shanghai Key Laboratory of Molecular Catalysis and Innovative Materials, Collaborative Innovation Center of Chemistry for Energy Materials, Shanghai Wusong Laboratory of Materials Science, State Key Laboratory of Porous Materials for Separation and Conversion, College of Smart Materials and Future Energy, Fudan University, China; Laboratory of Advanced Materials, Aqueous Battery Center, Shanghai Key Laboratory of Molecular Catalysis and Innovative Materials, Collaborative Innovation Center of Chemistry for Energy Materials, Shanghai Wusong Laboratory of Materials Science, State Key Laboratory of Porous Materials for Separation and Conversion, College of Smart Materials and Future Energy, Fudan University, China; Laboratory of Advanced Materials, Aqueous Battery Center, Shanghai Key Laboratory of Molecular Catalysis and Innovative Materials, Collaborative Innovation Center of Chemistry for Energy Materials, Shanghai Wusong Laboratory of Materials Science, State Key Laboratory of Porous Materials for Separation and Conversion, College of Smart Materials and Future Energy, Fudan University, China

**Figure 1.**
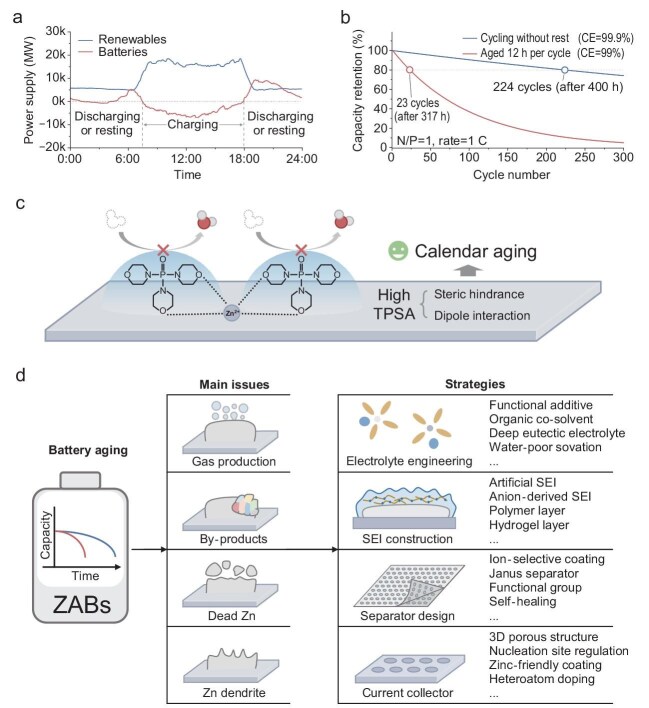
(a) Practical operating state of large-scale energy storage systems. Data acquired from CAISO on 7 April 2026. (b) Simulated degradation of ZABs at N/P ratio = 1 under laboratory ‘continuous cycling’ test (CE = 99.9%) and ‘aged 12 h per cycle’ condition (CE = 99%). (c) Schematic illustration of interfacial ionic environment remodeling by high-TPSA molecules to achieve superior calendar life. (d) Primary challenges of battery aging in ZABs and corresponding strategies.

The global transition toward a carbon-neutral energy landscape necessitates the large-scale integration of intermittent renewable energy sources such as solar and wind power. The intermittent output of these sources demands electrochemical energy storage systems that are both robust and flexible, capable of reliably buffering energy over extended timeframes (Fig. 1a) [1]. Metallic Zn-based aqueous batteries (ZABs) offer inherent safety, low cost, and high capacity, positioning them as ideal candidates for grid-scale energy storage [2]. However, recent reports reveal that while ZABs exhibit exceptional lifespan under uninterrupted short-term cycling, they suffer from rapid capacity fade and limited calendar life during intermittent cycling or resting states (Fig. 1b) [3]. This dilemma hinders ZAB-based energy storage systems from satisfying the demands of renewable energy integration and effective energy storage during inactive grid periods [4]. More importantly, this unavoidable calendar aging of ZABs in practical scenarios remains underexplored, and effective strategies to address the insufficient calendar life of ZABs are still very limited.

Unlike cycling aging, which is predominantly governed by faradaic charge-transfer kinetics and periodic lattice strain, calendar aging represents a spontaneous thermodynamic descent toward equilibrium during storage. For ZABs, the accumulation of active H_2_O/H_3_O^+^ at the electrode interface leads to irreversible corrosion processes, destroying the thermodynamic stability and reversibility of the electrode during both calendar aging and cycling process. In a recent study published in *National Science Review*, Yang *et al*. introduced topological polar surface area (TPSA) as a molecular descriptor, proving that

high-TPSA additives can effectively reshape the interfacial ionic environment to achieve superior calendar life and Coulombic efficiency of zinc anodes [5]. Their results show that trimorpholinophosphine oxide (TPO) featuring a high TPSA and multiple dipole moments, offers multiple Zn^2+^ binding sites (Fig. 1c). This topological polarity enhances Zn^2+^ coordination and strong surface adsorption, forming migration channels within the electrolyte-electrode interface and inducing interfacial ion redistribution. Furthermore, TPO alters the Zn^2+^ solvation structure through steric effects, creating a water-deficient interfacial layer on the zinc anode. These combined features substantially improve the calendar life and kinetic properties of batteries. Surprisingly, TPO-modified cells retain a high Coulombic efficiency of over 95% for an unparalleled 2500 hrs during intermittent aging, whereas unmodified cells show a precipitous decline in performance after a mere 72 hrs. Importantly, this study introduces two calendar aging protocols that directly simulate real‑world operating scenarios. Diverging from traditional uninterrupted cycling, this work highlights the critical role of the electrode-electrolyte interface in maintaining stability under static conditions. The intermittent aging test simulates routine grid dispatch to assess the battery’s capacity to recover from short-term idle periods. Meanwhile, the continuous incremental aging test simulates uncertain dormant durations caused by dispatch variations or seasonal standby, thereby evaluating capacity restoration after prolonged rest. These assessments transcend the limited insights of conventional cycling by directly examining whether the deposited zinc can still be cleanly and repeatedly stripped. This requirement aligns far more closely with the rigorous demands of practical large scale energy storage than any conventional continuous cycling protocol.

In prevailing works on ZABs, reports of Zn anode longevity exceeding thousands of cycles at high current densities are not infrequent [6]. Yet such metrics frequently obscure more than they illuminate. High-rate cycling often involves continuous stripping and plating that can dynamically clean the electrode surface or maintain a kinetic dominance over parasitic reactions. Conversely, calendar aging reveals the intrinsic thermodynamic and chemical compatibility of the Zn metal with its aqueous environment during electrochemical rest. It is worth noting that, regardless of whether the degradation originates from zinc corrosion at electrochemical resting or from the cumulative losses sustained during repetitive plating and stripping cycles, the focal point of battery aging resides at the electrode-electrolyte interface. Consequently, as shown in Fig. 1d, the rational design and manipulation of the solid-liquid interfacial properties between the electrolyte and Zn anode represents a fundamental prerequisite for improving both the calendar and cycling life of ZABs, a challenge that necessitates the synergistic optimization of multifaceted strategies.

Firstly, mitigating the parasitic consumption of the Zn metal by active H_2_O/H_3_O^+^ at the electrode interface through electrolyte engineering is a highly compelling strategy [7]. As the primary medium for interfacial reactions, regulating the solvation structure or hydrogen bond network of the aqueous electrolyte can effectively suppress the detrimental decomposition of these active species, thereby decelerating the calendar aging of the Zn anode. Representative strategies such as ‘water-in-salt’ or high concentration electrolytes exemplify this principle by confining water activity through extensive ion coordination. Nevertheless, the high cost and increased viscosity inherent to these high salt-to-solvent formulations severely compromise their scalability and practical adoption. Other compelling strategies involve the incorporation of functional additives or co-solvents, which can either disrupt the hydrogen bond network to minimize free water and preferentially adsorb onto the Zn surface. This surface adsorption is especially advantageous as it forms a molecular shield that limits the direct contact between active H_2_O/H_3_O^+^ and the Zn anode, thereby attenuating the corrosion rate and extending the calendar life of Zn anode.

If electrolyte engineering modulates the chemical potential driving interfacial reactions, interphase construction provides the physical barrier to mitigate such processes [8]. A compact and dense solid-electrolyte interphase (SEI) not only reduces the effective surface area available for corrosion but also fundamentally shifts the calendar aging mechanism from a linear regime controlled by surface reactions to a slower regime governed by diffusion [9]. For example, *in situ* formation of anion-derived or fluorinated SEI via electrolyte decomposition can create a thin and compact inorganic layer at the Zn anode interface. This SEI can suppress side reactions and enhance the interfacial kinetics of Zn^2+^ ion migration, resulting in improved rate performance and power density. *Ex situ* artificial SEI coatings such as tunable and programmable polymer- or hydrogel-based layers can provide better interfacial protection against dendrite formation and interfacial hydrogen evolution, but they are plagued by poor adhesion and potential delamination from the Zn anode. Bridging the gap between *in situ* and *ex situ* SEI strategies will be pivotal in mitigating the calendar aging of ZABs.

Furthermore, advanced designs of separators and current collectors represent critical yet underexplored determinants of calendar aging in ZABs. A separator characterized by highly uniform submicron pore distribution can homogenize the flux of Zn^2+^ ions arriving at the electrode surface, thereby promoting compact plating and suppressing dendrite formation. Functionalized separators can further extend this benefit by actively scavenging deleterious species, as exemplified by the incorporation of specific functional groups or ion-selective moieties that trap reactive components and thus inhibit undesirable parasitic reactions [10]. Similarly, carefully engineered current collectors can also exert comparable effects. By tuning the zincophilicity or three-dimensional architecture of the current collector, the nucleation sites and deposition morphology of Zn metal could be optimized, thereby promoting a more uniform Zn anode and an improved Coulombic efficiency.

In conclusion, this work moves beyond the conventional focus on cycle life alone, establishing calendar aging life as a critical parameter for assessing long-term stability of ZABs energy storage systems. Despite this advance, the calendar aging behavior of ZABs remains a largely uncharted domain. Future efforts should systematically evaluate the multi-dimensional impacts of ambient temperature, state-of-charge, N/P ratios, and electrolyte dosage on calendar life, as these variables may prove key to their long-term success.
